# Polydatin activates the Nrf2/HO-1 signaling pathway to protect cisplatin-induced hearing loss in guinea pigs

**DOI:** 10.3389/fphar.2022.887833

**Published:** 2022-08-04

**Authors:** Dafei Li, Haiyan Zhao, Piao Xu, Qiongping Lin, Tingting Zhao, Chubing Li, Zhong-Kai Cui, Guangyong Tian

**Affiliations:** ^1^ Department of Otorhinolaryngology-Head and Neck Surgery, The Third Affiliated Hospital of Southern Medical University, Guangzhou, China; ^2^ Guangdong Provincial Key Laboratory of Bone and Joint Degeneration Diseases, Southern Medical University, Guangzhou, China; ^3^ Department of Cell Biology, School of Basic Medical Sciences, Southern Medical University, Guangzhou, China

**Keywords:** polydatin, hearing loss, Nrf2, HO-1, cisplatin

## Abstract

Irreversible sensorineural hearing loss is one of the most common side effects after cisplatin treatment. Prevention and reversal of hearing loss caused by cisplatin are of great importance for cancer patients, especially children. Oxidative stress is an important cause of hearing loss resulted from cisplatin, unfortunately, there is no drug yet available that can completely prevent and reverse the ototoxicity from cisplatin. Polydatin (PD) possesses excellent antioxidant and anti-inflammatory effects, however, its role in the cisplatin-induced hearing loss has not been investigated. Herein, we have explored the preventive and therapeutic effects of PD on cisplatin-induced hearing loss and the possible underlying mechanisms. In the *in vivo* setting with guinea pigs, we have demonstrated that PD can reduce the threshold shift of auditory brainstem response (ABR) caused by cisplatin, promote the nuclear translocation of Nuclear factor erythroid-2 related factor 2 (Nrf2), increase the expression of Nrf2 and heme oxygenase-1 (HO-1), and thus reduce the loss of outer hair cells (OHCs). PD can ameliorate cisplatin-induced hearing loss through activating the Nrf2/HO-1 signaling pathway. This study provides a potential strategy for preventing and improving hearing loss resulted from cisplatin treatment in clinics.

## Introduction

Cisplatin is one of the effective chemotherapy drugs for a broad spectrum of tumors (Cepeda et al., 2007; Chovanec et al., 2017). Ototoxicity usually occurs after administration of cisplatin, manifesting permanent hearing loss and tinnitus (Theunissen et al., 2014; Gentilin et al., 2019; Watts, 2019). Side effects of cisplatin often lead to low quality of life, along with heavy family and social burdens (Clemens et al., 2018). Prevention and treatment of cisplatin-induced hearing loss are a major challenge, which clinicians and researchers around the world need to work together to tackle.

The mechanism of cisplatin-induced hearing loss remains unclear. Oxidative stress injury, inflammation, apoptosis and autophagy play an important role in the pathogenesis of cisplatin-induced hearing loss (Gentilin et al., 2019). Reactive oxygen species (ROS) production and antioxidant enzyme depletion are major molecular mechanisms of cisplatin-induced hearing loss (Sheth et al., 2017). The increase of NADPH oxidase and xanthine oxidase in cochlea after cisplatin exposure indicates the accumulation of ROS in cochlea (Lynch et al., 2005; Karasawa and Steyger, 2015). Accumulation of 4-hydroxynonenal, peroxynitrite and malondialdehyde also suggests lipid peroxidation in cochlea after cisplatin administration (Rybak, 2007; Park et al., 2019). High-dose cisplatin treatment can promote the production of a large number of ROS in cells, resulting in excessive consumption of antioxidant enzymes including superoxide dismutase (SOD), catalase, glutathione peroxidase (GSH-Px) and glutathione reductase, which ultimately leads to a sustained decline in intracellular antioxidant capacity (Sheth et al., 2017; Tang et al., 2021). ROS can also promote the production of inducible nitric oxide synthase (iNOS), which continuously promotes the production of NO, resulting in lipid peroxidation and hair cells damage (Watanabe et al., 2002; Li et al., 2006). Cisplatin induces the formation of caspase-3 by promoting oxidative stress injury, and leads to the apoptosis of hair cells, spiral ganglion neurons (SGNs), and vascular marginal cells in the organ of corti, eventually resulting in hearing loss (Gentilin et al., 2019). Strategies to combat cisplatin-induced ototoxicity include the use of small molecule drugs, improved administration methods and updated drug delivery systems (Yu et al., 2020). Antioxidant therapy is one of the main strategies to treat cisplatin ototoxicity (Tang et al., 2021). A large number of preclinical studies have shown that antioxidant drugs play an important role in reducing cisplatin-induced hearing loss, unfortunately, no drug yet can fully reduce cisplatin-induced ototoxicity without affecting the efficacy of cisplatin or causing other side effects (Yu et al., 2020). Finding novel and more effective drugs is of particularly importance in reducing and preventing cisplatin-induced hearing loss.

Polydatin (PD), also named piceid (3,4′,5-trihydroxystilbene-3-β-d-glucoside), is a natural active ingredient extracted from polygonum cuspidatum, a traditional Chinese medicinal plant. PD possesses the effects of anti-inflammatory, anti-oxidative stress, anti-ischemia injury, anti-atherosclerosis, anti-apoptosis, anti-tumor, anti-bacteria, and anti-virus, which has garnered considerable attention (Sun and Wang, 2020; Wu et al., 2020). PD is a precursor of resveratrol, and previous reports have demonstrated that resveratrol exhibited good antioxidant capacity in the inner ear, including inhibition of oxidative stress injury and autophagy (Seidman et al., 2013; Pang et al., 2019; Xiong et al., 2019). Resveratrol can reduce noise induced hearing loss and cochlear hair cell loss *via* enhancing the activity of sirtuin 1 in a mouse model (Xiong et al., 2017). After intragastric administration of PD and resveratrol in rats, the dynamic transformation of PD and resveratrol reached equilibrium *in vivo*, while PD was the dominant form. In addition, PD, compared with resveratrol, was more easily absorbed through oral administration, and presented better antioxidant effects *in vivo*, suggesting that PD may replace resveratrol as a clinical antioxidant (Wang et al., 2015).

The mechanisms of PD functions in different experimental settings were partially investigated. Through the Nrf2/HO-1 signaling pathway, PD was reported to improve the spinal cord injury in rats by inhibiting oxidative stress and microglial apoptosis (Lv et al., 2019b) and to ameliorate the radiation-induced testicular injury by reducing ROS (Ma and Jia, 2018), as well as to inhibit the expression of ROS and TGF-β1 in the airway of asthma, therefore reducing airway inflammatory response in a mouse model (Zeng et al., 2019). PD could also enhance sirtuin 1 activity, increase SOD2 protein expression and activity, reduce intestinal injury caused by oxidative stress during hemorrhagic shock, and inhibit intestinal apoptosis and histological changes (Zeng et al., 2015). Moreover, the anti-inflammatory effect of PD played an important role in reducing microglial inflammation and traumatic spinal cord injury by regulating iNOS and NLRP3 inflammasomes (Lv et al., 2019a). However, the mechanism of PD in protection and treatment of cisplatin-induced hearing loss has not yet been explored.

Nrf2, as an important transcription factor regulating cellular oxidative stress response, plays an important role in maintaining cellular redox homeostasis (Ma, 2013; Cuadrado et al., 2018). Nrf2 alleviates cell damage caused by oxidative stress and maintains the dynamic balance of redox system by inducing and regulating the expression of various antioxidant factors (Schmidlin et al., 2019). A large number of studies have shown that Nrf2 is a major regulatory factor in a variety of cell protection responses, and it is a key molecular node in specific disease groups, which provides a new strategic target for drug development and reuse (Cuadrado et al., 2019). In addition, experiments have proved that drugs can play a positive role in hearing protection by activating the Nrf2 signaling pathway, more details can be found in this review (Li et al., 2021).

In this study, we aimed to explore the effects of PD on cisplatin-induced hearing loss, and attempted to unravel the underlying mechanism. First, we evaluated the changes of Nrf2, HO-1, caspase-3 and iNOS in the cochlea before and after cisplatin administration with a guinea pig model. The expression of caspase-3 and iNOS in the cochlea was declined with the application of PD, which promoted the expression of Nrf2 and HO-1, thereby inhibiting oxidative stress injury caused by cisplatin. PD may become a new strategy to prevent and treat cisplatin-induced hearing loss by activating the Nrf2/HO-1 signaling pathway.

## Materials and methods

### Animals

In this study, male adult hartley guinea pigs (250 ∼ 350 g) were purchased from the Animal Experimental Center of Southern Medical University. Each guinea pig underwent auricle reflex and electric otoscope examination to exclude external auditory canal lesions and otitis media. Animals were kept in a temperature-controlled environment (23°C), fed under standard 12-h light/dark conditions with access to food and water, *ad libitum*. This experiment protocol was approved by the Animal Ethics Committee of the Third Affiliated Hospital of Southern Medical University and strictly abided by animal ethics norms. 56 Guinea pigs were randomly divided into the following eight groups, 1) control group, 2) cisplatin (CDDP) group, 3) solvent (2% DMSO in saline)+CDDP group, 4) 40 mg/kg PD group, 5) dexamethasone (Dex) +CDDP group, 6) 20 mg/kg PD + CDDP group, 7) 40 mg/kg PD + CDDP group, 8) 80 mg/kg PD + CDDP group, n = 7 for each group, respectively.

### Drug administration

PD (95%, cod P109978, Aladdin, America) solution, dexamethasone sodium phosphate injection (DEX, 1 ml: 5 mg, SINOPHARM, China) and cisplatin (2 mg/ml, Yunnan Phytopharmacy, China) were used in this study. 2% (v/v) DMSO in saline was used to dissolve PD. Control group received 1.0 ml of normal saline [intraperitoneal injection (i.p.)] every day for 3 days. PD group was administered with PD (40 mg/kg/day, i.p.) for 3 days. CDDP group was administered with cisplatin (12 mg/kg, i.p.) once and then normal saline (1.0 ml, i.p.) every day for 2 days. Solvent + CDDP group was administered with solvent 1 h before cisplatin (12 mg/kg, i.p.) was injected and then normal saline (1.0 ml, i.p.) every day for 2 days. PD group was administered with PD (40 mg/kg/day, i.p.) for 3 days. DEX group was administered with DEX (10 mg/kg, i.p.) 1 h before cisplatin (12 mg/kg, i.p.) was injected and then DEX (10 mg/kg, i.p.) every day for 2 days. 20 mg/kg PD + CDDP group was administered with PD (20 mg/kg, i.p.) 1 h before cisplatin (12 mg/kg, i.p.) was injected and then PD (20 mg/kg, i.p.) every day for 2 days. 40 mg/kg PD + CDDP group was administered with PD (40 mg/kg, i.p.) 1 h before cisplatin (12 mg/kg, i.p.) was injected and then PD (20 mg/kg, i.p.) every day for 2 days. 80 mg/kg PD + CDDP group was administered with PD (80 mg/kg, i.p.) 1 h before cisplatin (12 mg/kg, i.p.) was injected and then PD (80 mg/kg, i.p.) every day for 2 days. On the third day after drug administration, guinea pigs were anesthetized with 1% pentobarbital sodium at a dose of 40 mg/kg for evaluation.

### Auditory function evaluation

Auditory brainstem response (ABR) test was performed in a sound-proof electrical shielding room on an ABR recording system (Tucker-Davis Technologies). The recording electrode was placed in the middle of the cranial top at the midpoint of the upper edge of the bilateral auricle. The reference electrode was placed under the donor ear, and the grounding electrode was placed under the contralateral ear. ABR thresholds were measured with click and tone bursts at 4, 8, 16, 24, and 32 kHz, respectively. Stimulus level decreased in 10 dB steps and then in 5 dB steps near threshold. The hearing threshold is determined based on wave III and defined as the lowest stimulus level that elicited a reproduceable waveform with identifiable peaks. ABR threshold was detected 24 h before and 72 h after drug administration in each group.

### Cochlear pathology

After the guinea pigs were sacrificed by injection of excessive anesthetics, four cochleas in each group were harvested, fixed in 4% buffered paraformaldehyde for 12 h, and decalcified in 10% EDTA for 14 days, and embedded in paraffin according to the reported method (Bako et al., 2015). Samples were sectioned (5 μm in thickness) parallel to the long axis of the cochlea. Hematoxylin and eosin (H&E) staining was performed, and the general morphology of the cochlea, the organ of corti, basilar membrane, stria vascularis (SV) and SGNs in each cochlear turn were observed under an Olympus microscope (BX-51, Japan).

SGNs were counted in turn 1, turn 2, and turn 3 of sections. The relevant area of the Rosenthal’s canal was measured in digital photomicrographs of each canal profile. The circumference of the canal was traced with a cursor using the ImageJ (Bethesda, MD, United States). The software then calculated the area within the outline. SGN density was measured as the number of SGNs per mm^2^ (Park et al., 2019). One section was collected out of every five sections to avoid double counting of adjacent sections, and the average count resulted from the three sections represented the number of SGNs in the Rosenthal’s canal. Since turn 4 was located at the cochlear apex, it was difficult to obtain a continuous and full Rosenthal’s canal in the section, hence only the number of SGNs in turn 1, turn 2 and turn 3 were counted. We used four cochleas per group for SGNs counting.

### Scanning electron microscope analysis

Additional cochleas of the control group and CDDP group were taken for scanning electron microscopy (SEM) to observe the hair cells (HCs) damage. SEM of the cochlea follows these steps, cochlea dissection, fixation of the tissue at 2.5% glutaraldehyde, gradient dehydration of samples, cochlear microanatomy to expose the basilar membrane by removing the volute and tectorial membrane, critical point drying, gold sputter coating, and scanning using the Hitachi SU8100 electron microscope. We assessed the integrity of the HCs in the basilar membrane of the cochlea and the morphology of the stereocilia on the HCs.

### Staining of cochlear basilar membrane and OHCs count

Five cochleas in each group were harvested, fixed in 4% buffered paraformaldehyde for 12 h, and decalcified in 10% EDTA for 7 days. After 1 week of decalcification, the basilar membranes of each turn were carefully separated from the worm shaft and divided into four sections according to a line (the hypothetical line was made at the basal turn of the cochlea close to the round window to the apex of the cochlea). All the above operations were performed under an anatomical microscope (OLYMPUS, SZX12, Japan). After rinsing with PBS, sections were stained with FITC-phalloidin (1:100) for 30 min. The basilar membrane was carefully spread on the glass plate after rinsing with distilled water three times, and the excess water was dabbed with filter paper, and then the antifade mounting medium (AR0036, Boster, Wuhan, China) was added dropwise and sealed. Digital images were captured under a fluorescence microscope (Olympus, BX-51, Japan) and OHCs counts were performed using ImageJ (Bethesda, MD, United States).

### Immunohistochemistry

For immunostaining of Nrf2, HO-1, caspase-3 and iNOS, cochlear sections were rehydrated before the antigen retrieval process (0.01 M sodium citrate pH 6.0 for 2 min in the microwave oven). After washing with PBS, tissues were blocked in 5% normal goat serum for 30 min and in the diluted primary antibody (Nrf2 rabbit polyclonal, 16396-1-AP, 1:300, Proteintech, Wuhan, China; HO-1 rabbit polyclonal, 110701-1-AP, 1:300, Proteintech, Wuhan, China; caspase-3 rabbit polyclonal, 19677-1-AP, 1:500, Proteintech, Wuhan, China; iNOS rabbit polyclonal, 18985-1-AP, 1:500, Proteintech, Wuhan, China) overnight at 4°C. The next day, the slides of Nrf2 and HO-1 were washed extensively and appropriate fluorescently labeled secondary antibodies (Dylight 488 Goat Anti-Rabbit IgG, A23220, 1:300, Abbkine, California, America) were applied for 1 h at 37°C. Meanwhile, the slides of caspase-3 and iNOS were washed extensively and appropriate fluorescently labeled secondary antibodies (Dylight 649 Goat Anti-Rabbit IgG, A23220, 1:300, Abbkine, California, America) were applied for 1 h at 37 °C. All slides were then washed thoroughly and treated with DAPI (4′, 6-diamidino-2-phenylindole, C0065, Solarbio, Beijin, China) for DNA visualization. Cover slips were mounted with the antifade mounting medium (AR0036, Boster, Wuhan, China) to inhibit fluorescence quenching. Digital images were taken under a fluorescence microscope (OLYMPUS, BX-51, Japan).

### Western blot

Three dissected cochleas in each group were collected on ice, stored at −80°C, and lysed using a RIPA buffer (Biyotime, Shanghai, China). The concentration of the extracts was determined with the Micro BCA kit (Solarbio, Beijing, China). Protein samples were separated with sodium dodecyl sulfate polyacrylamide gel electrophoresis and transferred to a PVDF membrane (Millipore, MA, United States), which were blocked with 5% nonfat dry milk in TBS with 0.1% Tween-20. The membranes were incubated with primary antibodies, namely rabbit anti-Nrf2 (16396-1-AP, 1:1000, Proteintech, Wuhan, China), anti-HO-1 (110701-1-AP, 1:1000, Proteintech, Wuhan, China), anti-caspase-3 (19677-1-AP, 1:500, Proteintech, Wuhan, China), anti-iNOS (18985-1-AP, 1:500, Proteintech, Wuhan, China) and anti-β-actin (WL01372, 1:1000, Wanleibio, Shenyang, China) overnight. After incubation with the secondary antibody (ZB-2306, 1:5000, ZSGB-BIO, Beijing, China), and extensive membrane washing, the blots of immune reactivity were illustrated through enhanced chemiluminescence (KF001, Affinity Biosciences, OH, United States). ImageJ (Bethesda, MD, United States) was used for densitometric comparisons. β-actin density measurements were used as loading controls.

### Statistical analyses

GraphPad Prism 8.0 software (GraphPad Software Inc., San Diego, CA, United States) was used for statistical analysis. Data were presented as mean ± standard error of the mean (SEM). Multiple comparisons were assessed using one-way or two-way analysis of variance (ANOVA), and the analysis of variances followed by Tukey’s *post hoc* test was employed. *p <* 0.05 was considered statistically significant.

## Results

### Polydatin can reduce hearing loss induced by cisplatin

**FIGURE 1:**
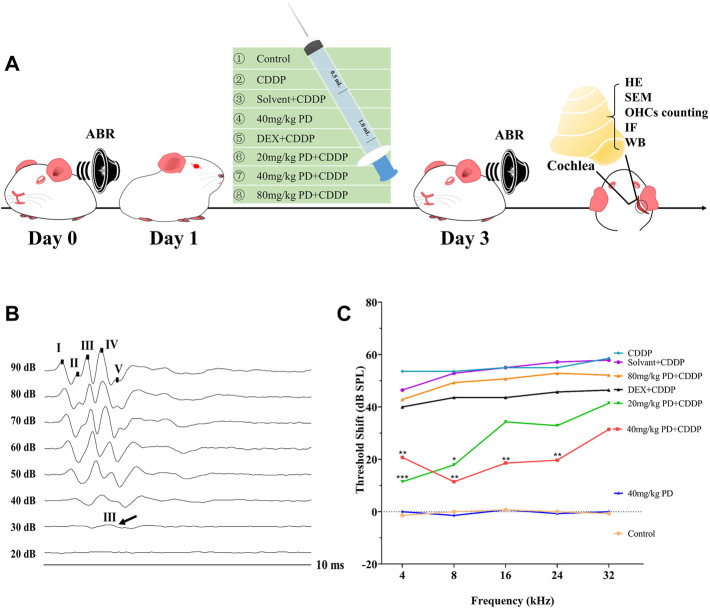
PD reduces the ABR threshold shift induced by cisplatin in guinea pigs. **(A)** Experimental groups and experimental operations taken at corresponding time points. **(B)** These are representative plots of the ABR detection of the control group. Wave III was the most prominent and stable in the ABR detection. **(C)** Comparison of ABR threshold shift among each group. PD (40 mg/kg) showed good hearing protection at 4, 8, 16, and 24 kHz after injection of CDDP, which was better than DEX (10 mg/kg, multi-dose intraperitoneal administration). PD alone had no effect on hearing in guinea pigs. *n* = 7. Data were presented as mean ± SEM. *p < 0.05, **p < 0.01, and ***p < 0.001, all versus the CDDP group.

In order to investigate the effect of PD on auditory function, we recorded the ABR threshold and threshold shift of eight different groups 24 h before and 72 h after cisplatin administration (Figure 1A). The selection of wave for the ABR thresholds depends on species, laboratory equipment, environment, etc. In our experimental setting, wave III was the most prominent and stable, therefore wave III was selected as the reference for ABR threshold judgment (Figure 1B). The ABR threshold at 4, 8, 16, 24, and 32 kHz were basically the same in each group before administration (Supplementary Figure S1). There was no significant difference in the ABR threshold between the control group and the 40 mg/kg PD group before and after administration of physiological saline and 40 mg/kg PD (Supplementary Figure S1). However, after cisplatin administration, ABR thresholds of the corresponding drug group were increased at all the above frequencies. The ABR thresholds of the CDDP group and solvent + CDDP group increased the most, and the ABR thresholds at high frequencies were higher than that at low frequencies after cisplatin administration. In order to study the hearing protection effect of PD, we set up three doses of PD, 20 mg/kg, 40 mg/kg and 80 mg/kg. The results showed that the ABR thresholds of guinea pigs at each frequency was lower than that of the CDDP group after the administration of 20 mg/kg PD, and the hearing protection effect at 4 and 8 kHz was the most prominent with statistically significant difference (Supplementary Figure S1). In addition, we found that PD (40 mg/kg) effectively mitigated the effects of cisplatin induced hearing loss. The ABR thresholds increase induced by cisplatin was significantly lower than that of the CDDP group and 20 mg/kg PD + CDDP group after treatment with PD (40 mg/kg), which exhibited protective effect on hearing at 4, 8, 16, and 24 kHz (Supplementary Figure S1). When the concentration of PD was increased to 80 mg/kg, the average ABR thresholds at each frequency was lower than that of the CDDP group, however, the difference was not statistically significant, suggesting that higher concentration of PD did not present additional protective effect on cisplatin induced hearing loss in guinea pigs. Meanwhile, in order to objectively evaluate the hearing protection effect of PD, we also employed the DEX group as the positive group according to the reported experimental results (Sun et al., 2016). Guinea pigs dosed with DEX (10 mg/kg, multi-dose administration) had lower mean ABR thresholds at all frequencies than that of the CDDP group, while the differences were not statistically significant. For assessment of the hearing changes before and after drug administration, we also calculated the mean ABR threshold shift of each group after drug administration (Figure 1C). In our experiment, after cisplatin injection, the mean ABR threshold shift increased significantly (53.57 ± 3.78, 53.57 ± 7.48, 55.00 ± 8.16, 55.00 ± 4.08, and 58.57 ± 3.78 dB at 4, 8, 16, 24, and 32 kHz, respectively). However, after PD (40 mg/kg) administration, the mean ABR threshold shift of guinea pigs decreased significantly. The administration of PD (40 mg/kg) exhibited significant protective effect on low-frequency hearing (20.71 ± 12.39, 11.43 ± 13.14, 18.57 ± 13.76, and 19.29 ± 13.67 dB at 4, 8, 16, and 24 kHz, respectively), however, little protective effect on hearing at 32 kHz (31.43 ± 15.73 dB, *p* = 0.053). Overall, our results suggest that PD (40 mg/kg) has the best hearing protection efficacy in our experimental animal model.

### Polydatin can reduce hair cell damage induced by cisplatin

**FIGURE 2:**
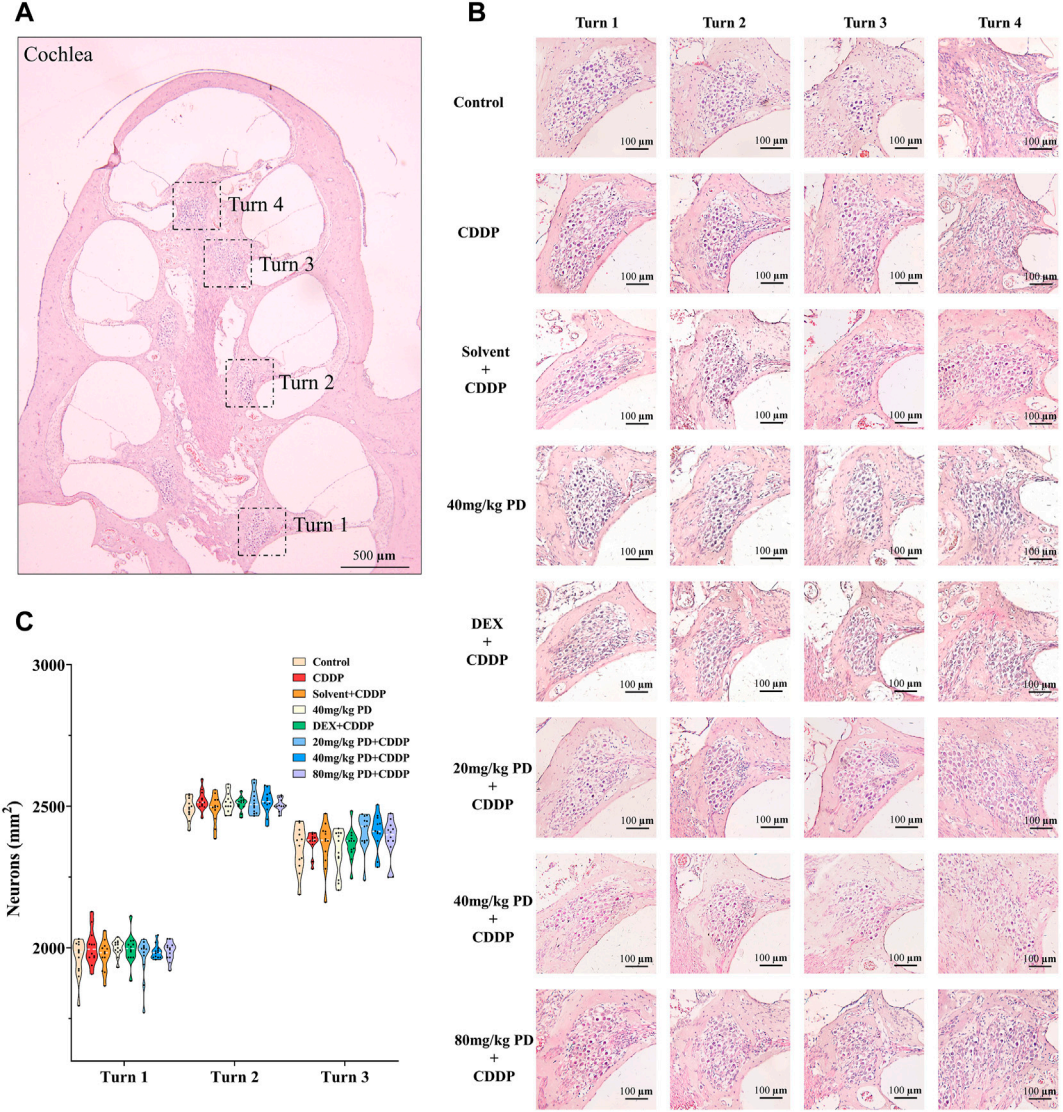
There was no significant loss of SGNs in guinea pig cochlea within 72 h after cisplatin or PD administration. **(A)** Paraffin sections (5 μm in thickness) were cut parallel to the longitudinal axis of the cochlea and then H&E staining was performed. According to the results, the cochlea of guinea pigs could be divided into turn 1, turn 2, turn 3, and turn 4. Scale bar, 500 μm. **(B)** In each group, SGNs in turn 1, turn 2 and turn 3 were closely distributed and no obvious loss was observed. Scale bar, 100 μm. **(C)** Graph shows the number of SGNs in the full Rosenthal’s in turn 1, turn 2 and turn3 in each group, *n* = 4. Data are presented as mean ± SEM.

For the purpose of further exploring the protective effect of PD on hearing loss, we observed the morphological changes of the cochlea with H&E staining (Figure 2A). The number of SGNs is an important morphological indicator for evaluating cisplatin ototoxicity, therefore, we counted the number of SGNs. In our experiment, we observed no obvious loss of SGNs in the full Rosentha’s canal (Figure 2B). In addition, the numbers of SGNs in turn 1, turn 2 and turn 3 were similar in each group (Figure 2C), suggesting that single dose cisplatin injection (12 mg/kg) did not cause significant loss of SGNs in a short period of time (72 h).

**FIGURE 3:**
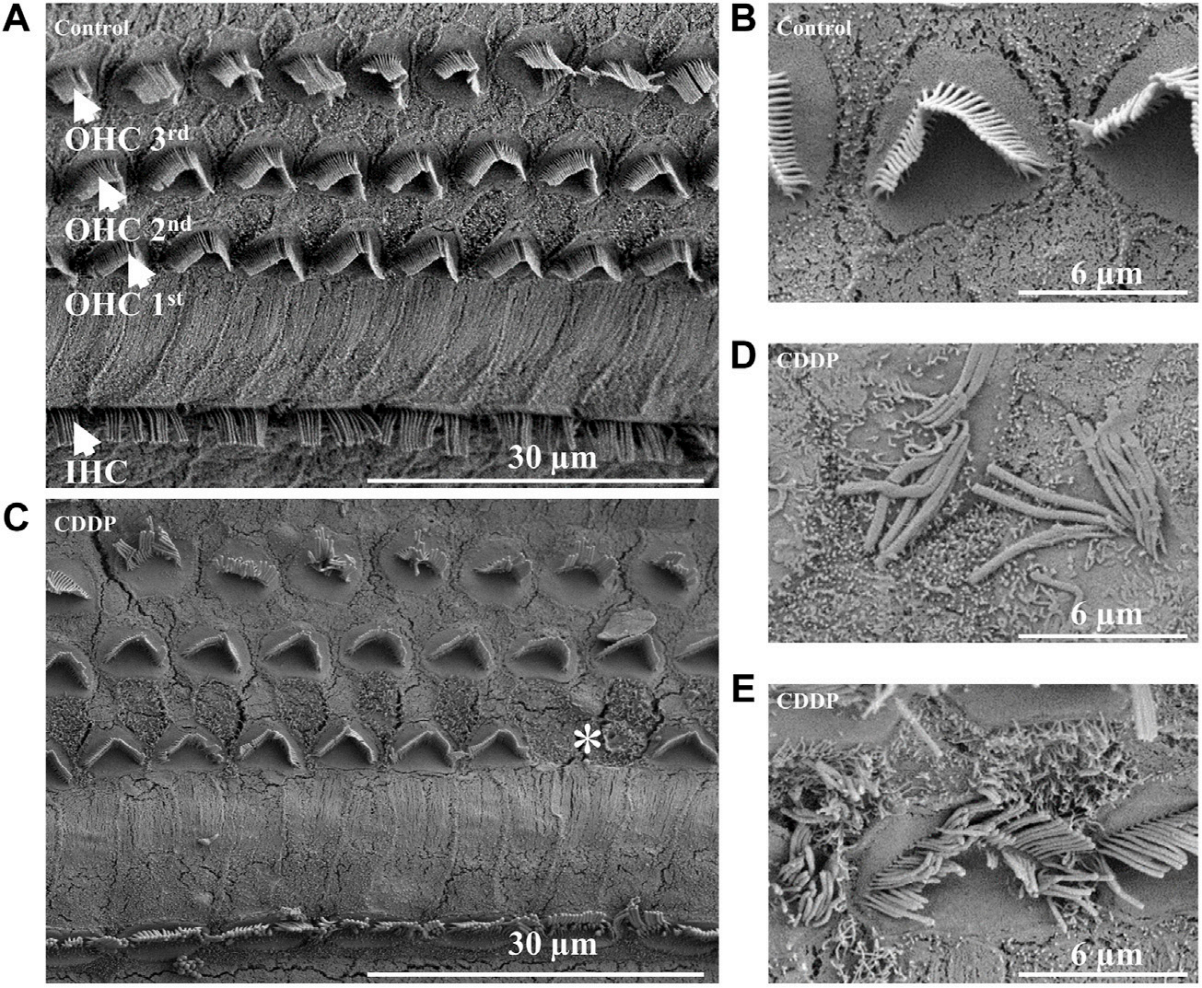
Cisplatin causes damage and loss of OHCs. **(A)** and **(B)** The IHCs and OHCs in the control group were arranged neatly without cell damage or loss. **(C–E)** In the CDDP group, loss of OHCs, abnormal arrangement, lodging and fusion of stereocilia were observed. Scale bar, 30 μm or 6 μm *represents missing OHCs.

In order to further explore the protective effect of PD on auditory function, we evaluated the survival of HCs on the basilar membrane. First, SEM of the cochlea was used to determine the damage of HCs after cisplatin administration. In our results, HCs on the basilar membrane of the control group were arranged neatly, without loss of cells (Figure 3A), and the cilia on the surface of the hair cells were straight, arranged in the inverted V shape (Figure 3B). Whereas, OHCs were lost in the CDDP group, and the arrangement of stereocilia on the remaining OHCs was disordered, presenting fusion and lodging (Figures 3C–E). Moreover, over the 72 h after cisplatin administration, no obvious damage of IHCs was observed by SEM.

**FIGURE 4:**
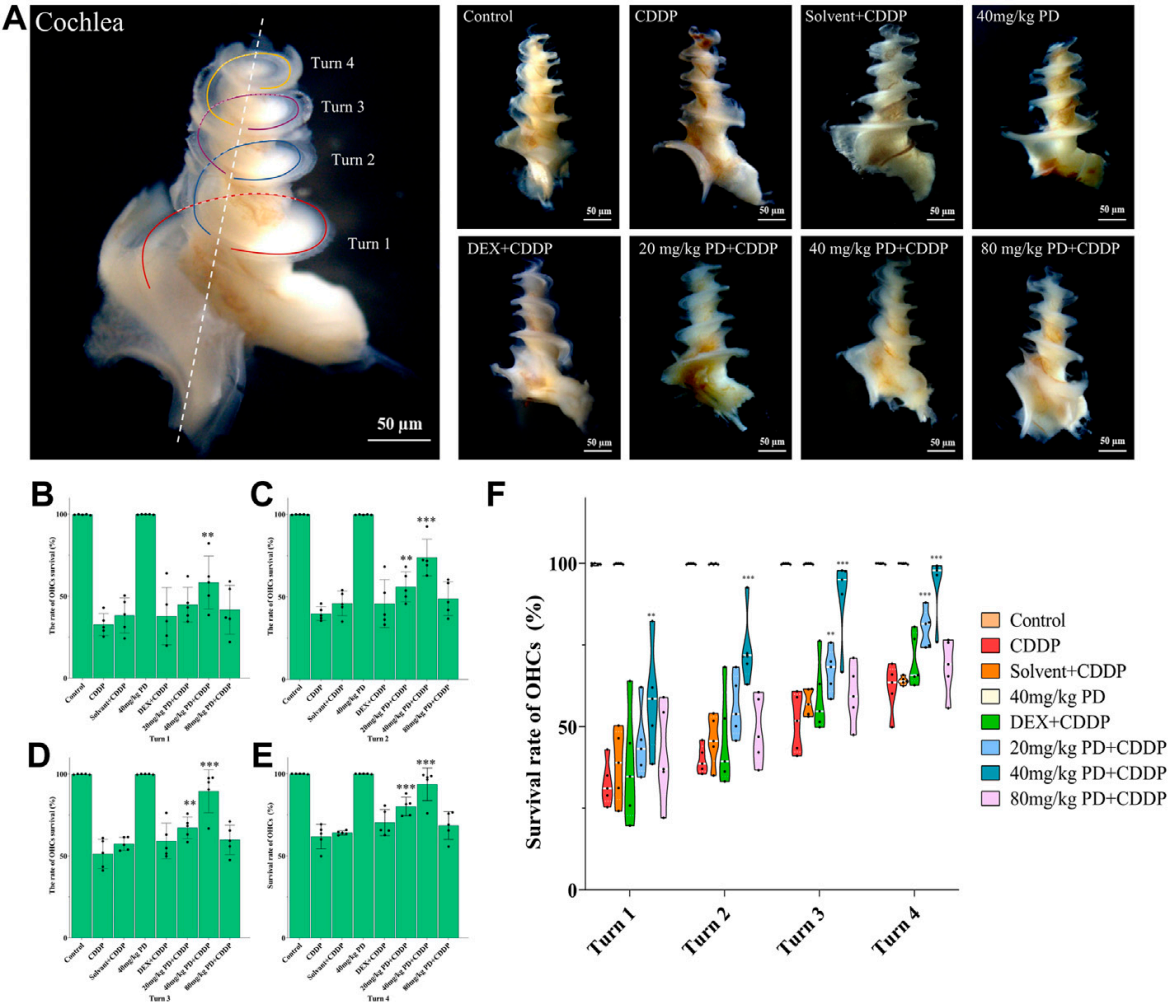
PD could reduce OHCs loss after cisplatin administration. **(A)** The cochlea of guinea pigs was divided into 4 turns, and the basilar membrane was dissected from them, and then stained and counted. Scale bar, 50 μm. **(B)** In turn 1, only the 40 mg/kg PD + CDDP group could improve the survival rate of OHCs compared with that of the CDDP group, *p* = 0.001. **(C)**, **(D)** and **(E)** In turn 2, turn 3, and turn 4, compared with the CDDP group, both 20 mg/kg PD + CDDP and 40 mg/kg PD + CDDP groups could improve the survival rate of OHCs, and the 40 mg/kg PD + CDDP group presented a better protective effect on OHCs than that of the 20 mg/kg PD + CDDP group (*p* = 0.003, *p* < 0.001 and *p* = 0.002 for turn 2, turn 3 and turn 4, respectively). **(F)** Graph shows the survival rate of OHCs after drug administration in each group. Either cisplatin or PD, the survival rate of OHCs always increases gradually from turn 1 to turn 4. *n* = 5. Data are presented as mean ± SEM. **p < 0.01, and ***p < 0.001, all versus the CDDP group.

**FIGURE 5:**
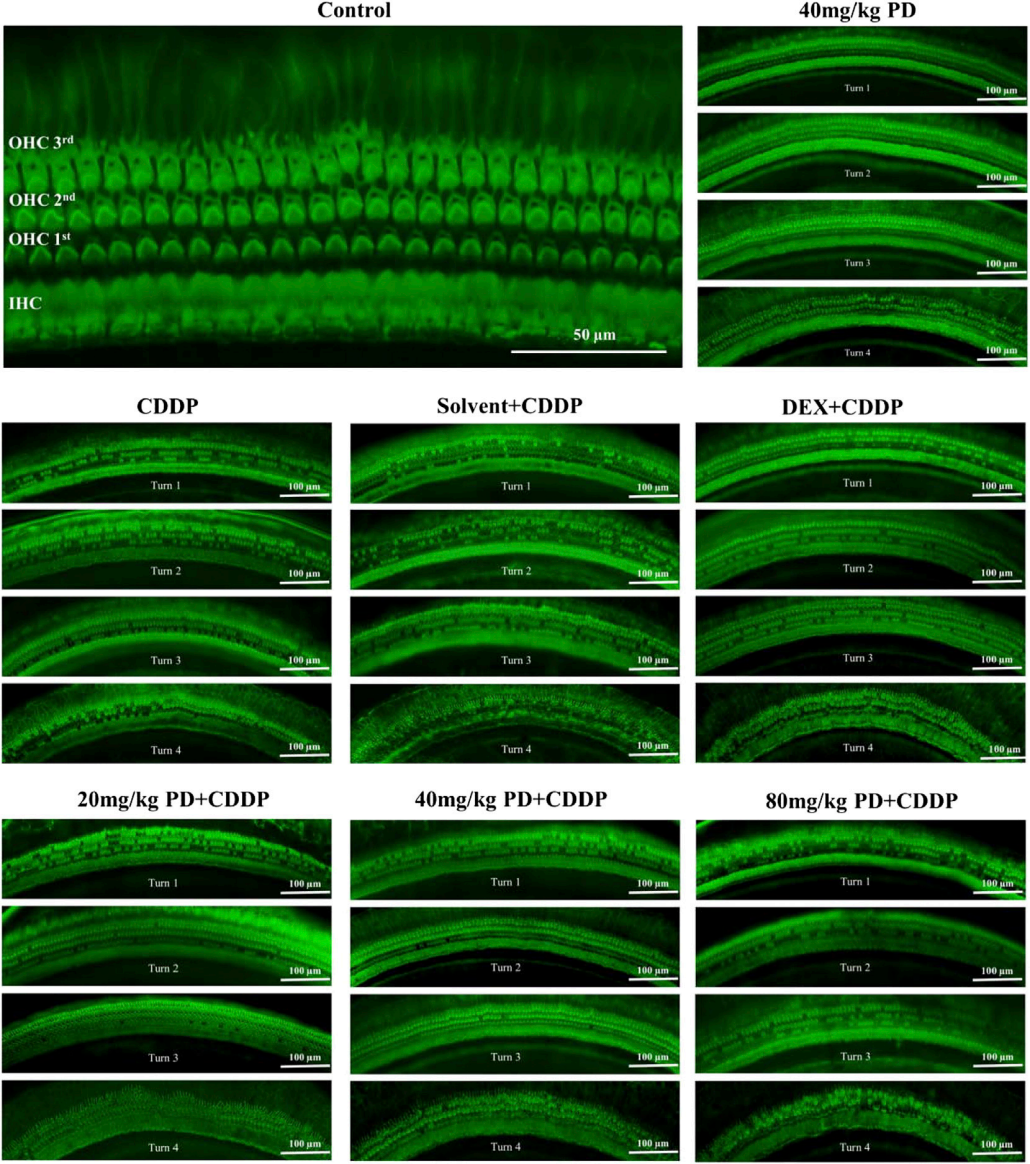
Survival of OHCs on the basilar membrane of each group after FITC-Phalloidin staining. In accordance with the SEM results, three layers of OHCs on basilar membrane were arranged successively, and there was another layer of IHCs near the cochlear axis. Surviving OHCs showed a green club-like structure, and the stereocilia on them showed a green-highlighted inverted V shape. Lost OHCs did not stain on the basilar membrane. In addition, no significant loss of IHCs was observed in all groups. *n* = 5. Scale bar, 50 μm or 100 μm.

We dissected the basilar membrane of cochlea and stained with FITC-phalloidin then observed and counted HCs under fluorescence microscope, so as to further explore whether the damage of HCs in each group matched the hearing loss and whether PD played a protective role in hearing by alleviating the damage of HCs. In the gross anatomy of the guinea pig cochlea, the basilar membrane of the cochlea can be divided into four turns, turn 1, turn 2, turn 3 and turn 4, which correspond to red, blue, purple and yellow respectively (Figure 4A). Observation and counting were carried out at the turn 1, turn 2, turn 3 and turn 4 of the basilar membrane with a fluorescence microscope. In the CDDP group, significant loss of OHCs occurred after cisplatin administration, and the survival rate of OHCs decreased gradually from the apex to the basal turn of the cochlea (61.74% ± 7.45%, 51.23% ± 8.89%, 39.85% ± 4.16%, and 32.69% ± 6.73% at turn 4, turn 3, turn 2 and turn 1, respectively) (Figures 4B–E). The survival rate of OHCs in the solvent + CDDP group was similar as that in the CDDP group. In addition, there was no significant loss of OHCs when PD (40 mg/kg) was injected alone without cisplatin. In the positive control group, after DEX administration (10 mg/kg, multi-dose intraperitoneal administration), the survival rate of OHCs in each turn was slightly higher than that in the CDDP group, however, the difference was not statistically significant. Most importantly, the survival rate of OHCs was significantly improved after PD administration. The survival rate of OHCs in turn 4 (80.09% ± 5.68%), turn 3 (67.13% ± 6.56%) and turn 2 (56.12%% ± 9.16%) was improved after the administration of PD (20 mg/kg) compared with that of the CDDP group (Figures 4C–E). Moreover, PD (20 mg/kg) did not protect outer hair cells at turn 1 (Figure 4B). Compared with PD (20 mg/kg), PD (40 mg/kg) could significantly improve the survival rate of OHCs after cisplatin administration (93.60% ± 9.96%, 89.51% ± 13.10%, 73.83% ± 11.17%, and 58.34% ± 16.14% at turn 4, turn 3, turn 2 and turn 1) (Figures 4B–E). As the dose of PD increased, PD (80 mg/kg) did not further improve the survival rate of OHCs. We also found that the loss of the three layers of OHCs on the basilar membrane always started from the third row, followed by the second row and the first row, of which the third row was the most remarkable (Figure 5). Exactly like hearing loss caused by cisplatin, OHCs loss also shifts from a high-frequency position to a low-frequency position, ranged as turn 1 > turn 2 > turn 3 > turn 4 (Figure 4F).

### Antioxidant therapeutic effect of Polydatin

**FIGURE 6:**
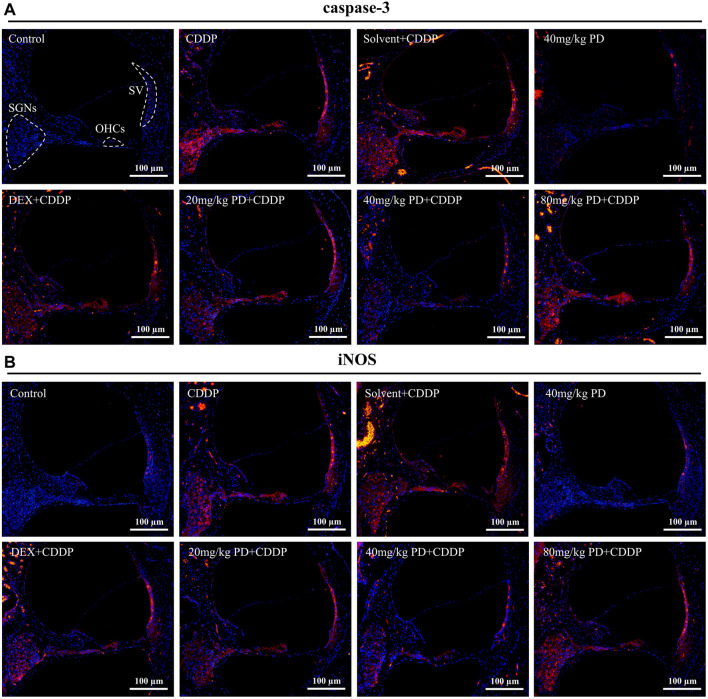
PD reduces oxidative stress injury and apoptosis induced by cisplatin. Representative immunofluorescence labelling in the cochlea for caspase-3 [red, **(A)**] and iNOS [red, **(B)**], double stained with DAPI in blue. The three dotted areas are SGNs, OHCs and SV. **(A,B)** Under normal circumstances, caspase-3 and iNOS were barely expressed in SGNs, OHCs and SV. However, apoptosis and oxidative stress damage occurred in cells in the three regions after cisplatin administration, accompanied by high expression of caspase-3 and iNOS, and marked increase in red fluorescence intensity. PD and DEX before cisplatin administration reduced oxidative stress damage and cell apoptosis in the cochlea, showing that the red fluorescence intensity was lower than that of the CDDP group. Scale bar, 100 μm.

Oxidative stress injury in cochlea is an important pathway of ototoxicity induced by cisplatin, we performed immunostaining of antioxidant factors (Nrf2 and HO-1), apoptosis markers (caspase-3) and iNOS to investigate the mechanism of PD in hearing protection. Cisplatin induced hearing loss is often accompanied by apoptosis of SGNs and HCs in the cochlea. Therefore, we first detected the expression of caspase-3 (Figure 6A). In the control group, there was little red fluorescence in SGNs, OHCs and Stria Vascularis (SV), indicating low caspase-3 expression in these regions. The same results were observed in the 40 mg/kg PD group injected with only PD. However, in the CDDP group and the solvent + CDDP group, these three regions showed intensified red fluorescence, indicating that apoptosis of SGNs, OHCs and SV occurred after cisplatin injection. Most importantly, after PD administration, the red fluorescence intensity in the three regions was lower than that in the CDDP group. Among them, 40 mg/kg PD + CDDP group was the most effective, and its fluorescence intensity was lower than that in the positive control group (DEX + CDDP group). In addition, ROS can also promote the production of iNOS, thus continuously promoting the production of NO, leading to lipid peroxidation and hair cell damage, hence, we also detected the expression of iNOS. Our results demonstrated the fluorescence expression of iNOS was consistent with caspase-3, and PD (40 mg/kg) could significantly reduce the production of iNOS induced by cisplatin (Figure 6B). The hearing protection effect ranged, PD (40 mg/kg) > PD (20 mg/kg) > DEX (10 mg/kg, multi-dose) > PD (80 mg/kg). PD (40 mg/kg) alone did not cause apoptosis and oxidative stress injury. In addition, the changes of ROS, RNS and NADPH oxidase in each group of cochlea were assessed after administration. PD (40 mg/kg) decreased the production of ROS and RNS and the activity of NADPH oxidase in cochlea after cisplatin administration (Supplementary Figure S2).

**FIGURE 7:**
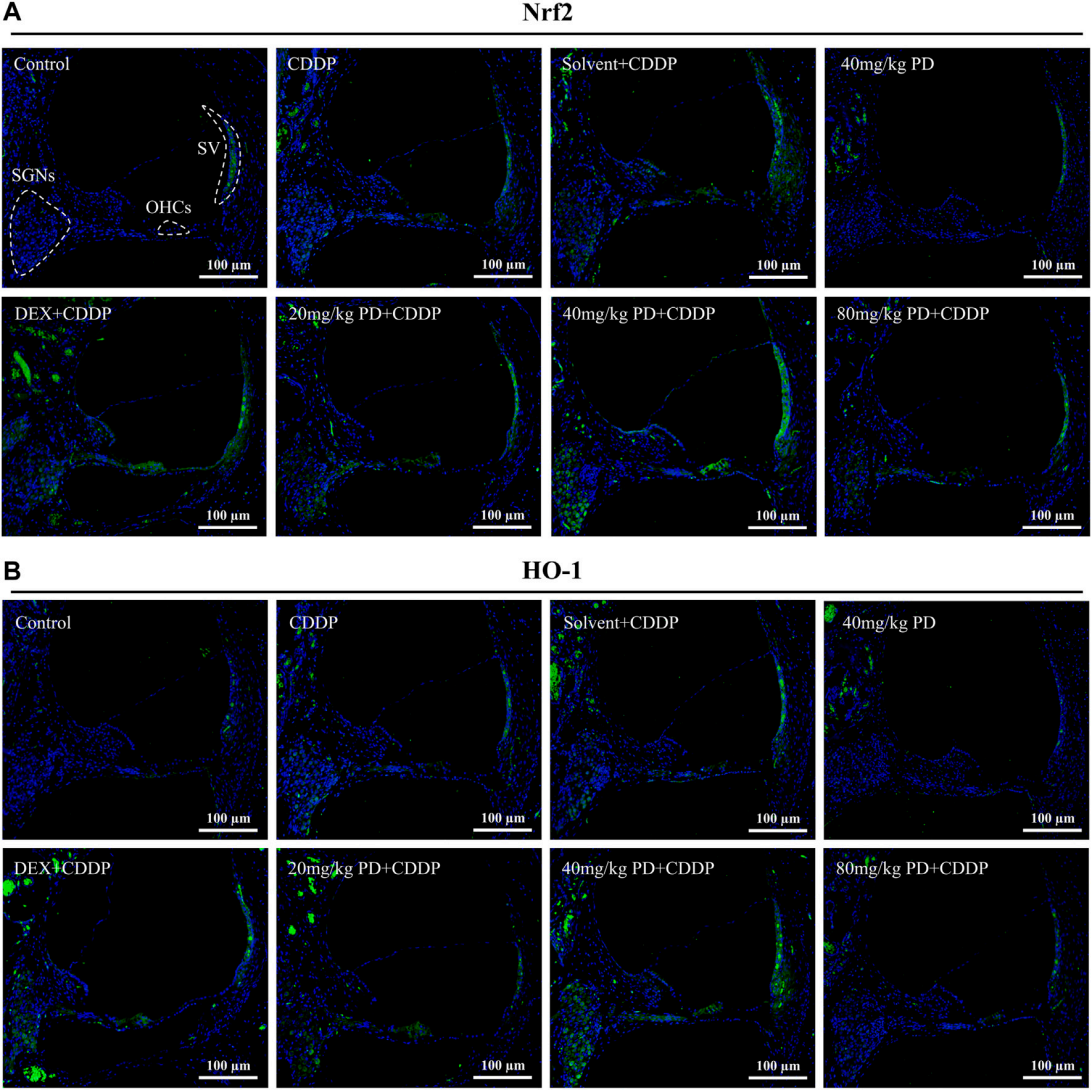
PD increases the antioxidant capacity of cochlea after cisplatin administration. Representative immunofluorescence labelling in the cochlea for Nrf2 (green, **(A)** and HO-1 (green, **(B)**, double stained with DAPI in blue. The three dotted areas are SGNs, OHCs and SV. **(A)** and **(B)** In the control group, Nrf2 and HO-1 were slightly expressed in SGNs, OHCs and SV. After cisplatin administration, Nrf2 translocation into the nucleus was increased, along with the increased expression of Nrf2 and HO-1, and the green fluorescence intensity was slightly increased. The expression of Nrf2 and HO-1 was increased by PD and DEX before cisplatin administration, and the green fluorescence intensity was higher than that of CDDP group. Scale bar, 100 μm.

We further detected the expression of Nrf2 (Figure 7A). In the control group, extremely weak green fluorescence could be observed in SGNs, OHCs and SV, indicating low expression of Nrf2 without external stimulation. The same situation also occurred in the 40 mg/kg PD group, suggesting that injection of PD alone would not improve the expression of Nrf2. In contrast, in the CDDP group and the solvent + CDDP group, the green fluorescence of these three regions was slightly increased, indicating that the expression of Nrf2 in SGNs, OHCs and SV increased after cisplatin injection. Moreover, after PD administration, the green fluorescence intensity in the three regions was higher than that in the CDDP group. Among them, 40 mg/kg PD + CDDP group was the most obvious, and its fluorescence intensity was higher than the positive group (DEX + CDDP group). HO-1 is an antioxidant regulated by Nrf2. In our results, the fluorescence expression of HO-1 in each group was similar as that of Nrf2 in each group. The hearing protection effect ranged, PD (40 mg/kg) > PD (20 mg/kg) > DEX (10 mg/kg, multi-dose) > PD (80 mg/kg). PD (40 mg/kg) alone did not significantly increase Nrf2 and HO-1 expression, while PD (40 mg/kg) could significantly improve the expression of HO-1 in the cochlea after cisplatin administration (Figure 7B). Similarly, the expression changes of Nrf2 downstream antioxidant factors in cochlea were detected by enzyme-linked immunosorbent assay. PD (40 mg/kg) promoted SOD1 expression and NQO1 activity after cisplatin administration (Supplementary Figure S3).

**FIGURE 8:**
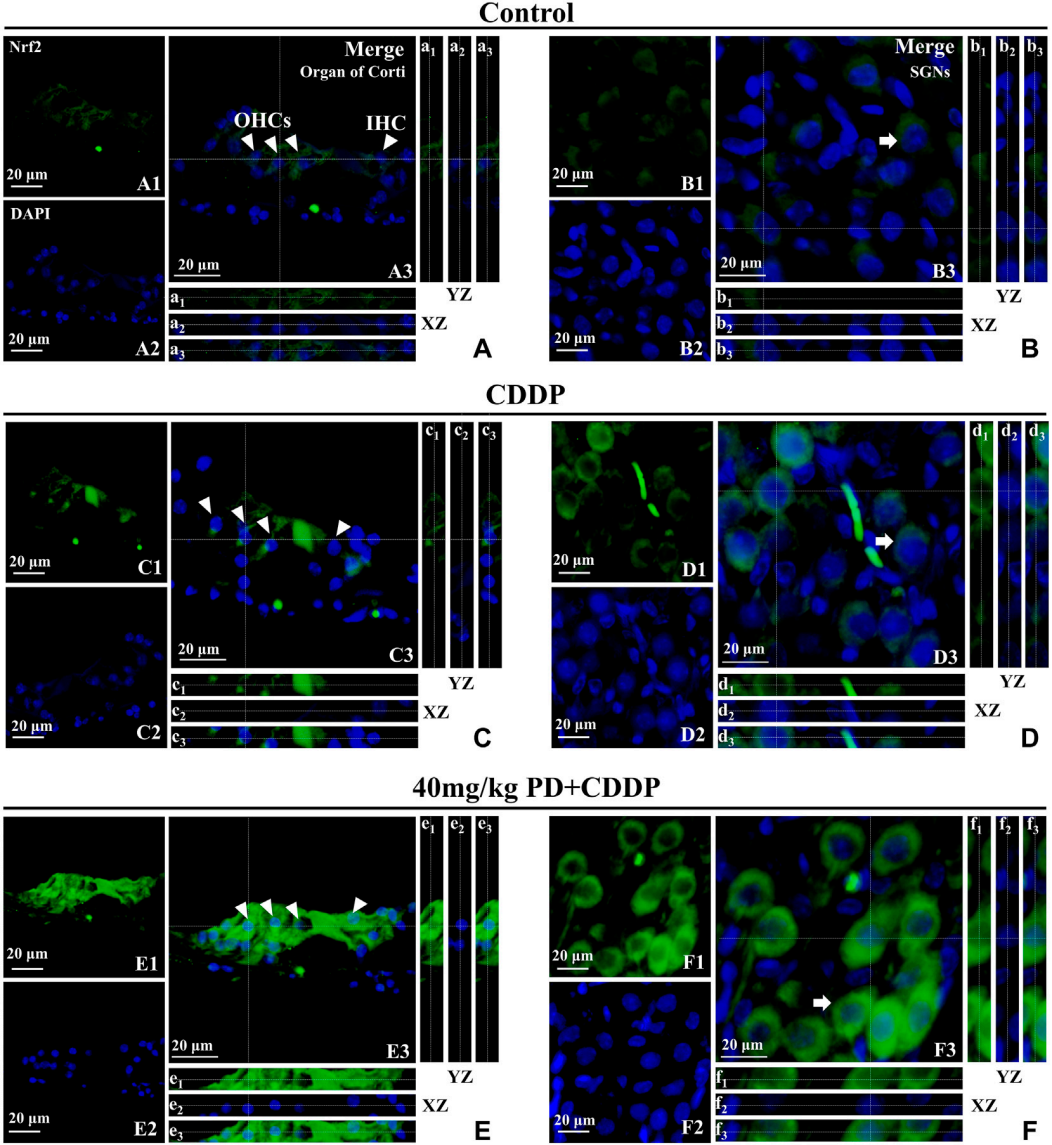
PD promotes Nrf2 translocation into the nucleus and increases HO-1 expression. Arrowheads indicate OHCs and IHC; arrows indicate SGNs. **(A–F)**: representative immunofluorescence labelling in the organ of corti (left panel) and SGNs (right panel) for Nrf2 (green), double stained with DAPI in blue. A3-F3 represent the merged images. X–Z and Y–Z cross-sections in boxes (referred to the dashed lines) from the Z-stack acquisitions show cytosolic or nuclear fluorescence signal (XZ and YZ boxes, a1-f1: Nrf2 fluorescence; a2-f2: DAPI staining; a3-f3: Merge). Cisplatin administration **(C,D)** induced an antioxidant response in the cochlea, resulting in a slight increase of Nrf2 expression in the cytoplasm [XZ and YZ c1-c3 and d1-d3 in **(C,D)**] compared to the control group [XZ and YZ A1-A3 and B1-B3 in **(A,B)**]. After PD administration **(E,F)**, the expression of Nrf2 was further enhanced in the cytoplasm and Nrf2 translocated to the nucleus [XZ and YZ e1-e3 and f1-f3 in **(E,F)**]. Scale bar, **(A–F)**, 20 µm.

We focused on the defense mechanism of Nrf2/HO-1 in order to gain a deeper understanding of the molecular mechanism of PD on hearing protection. In the control group, Nrf2 was slightly expressed in the cytoplasm (Figure 8A), as shown by the XZ cross-sections from the Z-stack acquisitions (boxes a1-a3 and b1-b3 in Figures 8A,B). In the CDDP group, the green fluorescence intensity of Nrf2 was weakly enhanced (Figures 8C,D), and slight nuclear accumulation of Nrf2 in OHCs and SGNs was observed by Z-stack analysis (boxes c1-c3 and d1-d3 in Figures 8C,D). Meanwhile, in the CDDP group, the increased expression of HO-1 in the cytoplasm was also observed in Figure 7. Moreover, the expression of Nrf2 was further increased after PD treatment (Figures 8E,F). Indeed, in HCs and SGNs, the green fluorescent signal of Nrf2 was detected not only in the cytoplasm but also in the nucleus, as clearly shown in the XZ cross-sections (boxes e1–e3 and f1–f3 in Figures 8E,F). In parallel, HO-1 expression was also increased in the same group (Figure 7). Altogether, Nrf2 and HO-1 expression increased in cisplatin affected cochlea after PD treatment, and Nrf2 promoted HO-1 expression by translocation into the nucleus.

**FIGURE 9:**
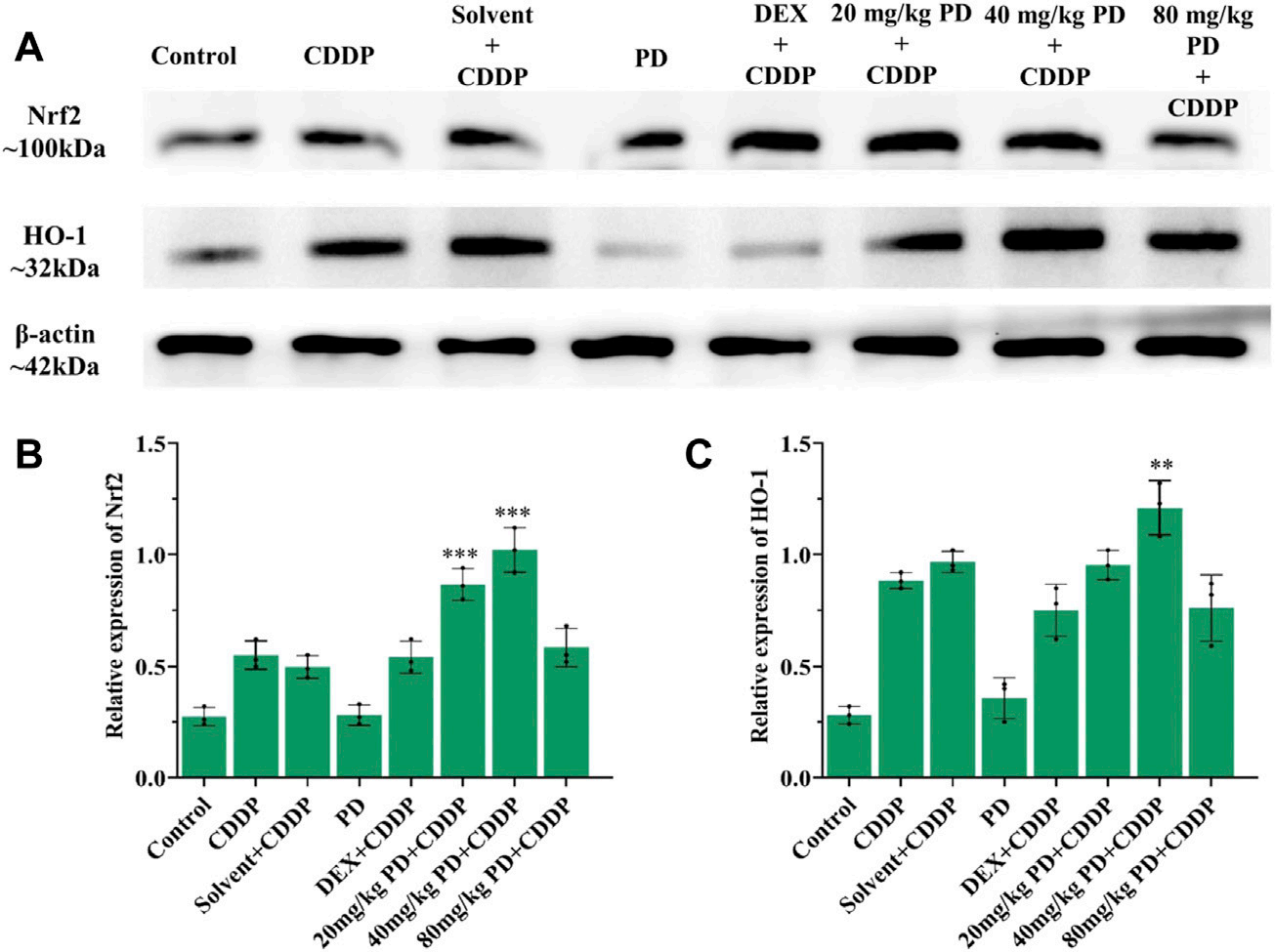
PD promotes the activation of Nrf2/HO-1 signaling pathway in the cochlea after cisplatin administration. **(A)** The expression of Nrf2 and HO-1 in each group after 3 days of drug administration. **(B)** and **(C)** PD administration before cisplatin increased the expression of Nrf2 and HO-1 in a concentration-dependent manner, presenting as PD (40 mg/kg) >PD (20 mg/kg) > PD (80 mg/kg). The effect of PD (40 mg/kg) was better than that of DEX (10 mg/kg, multi-dose) (*p <* 0.001). PD (40 mg/kg) injection alone did not improve the expression of Nrf2 and HO-1. *n* = 3. **p < 0.01, ***p < 0.001, all versus the CDDP group. Error bars represent mean ± SEM.

**FIGURE 10:**
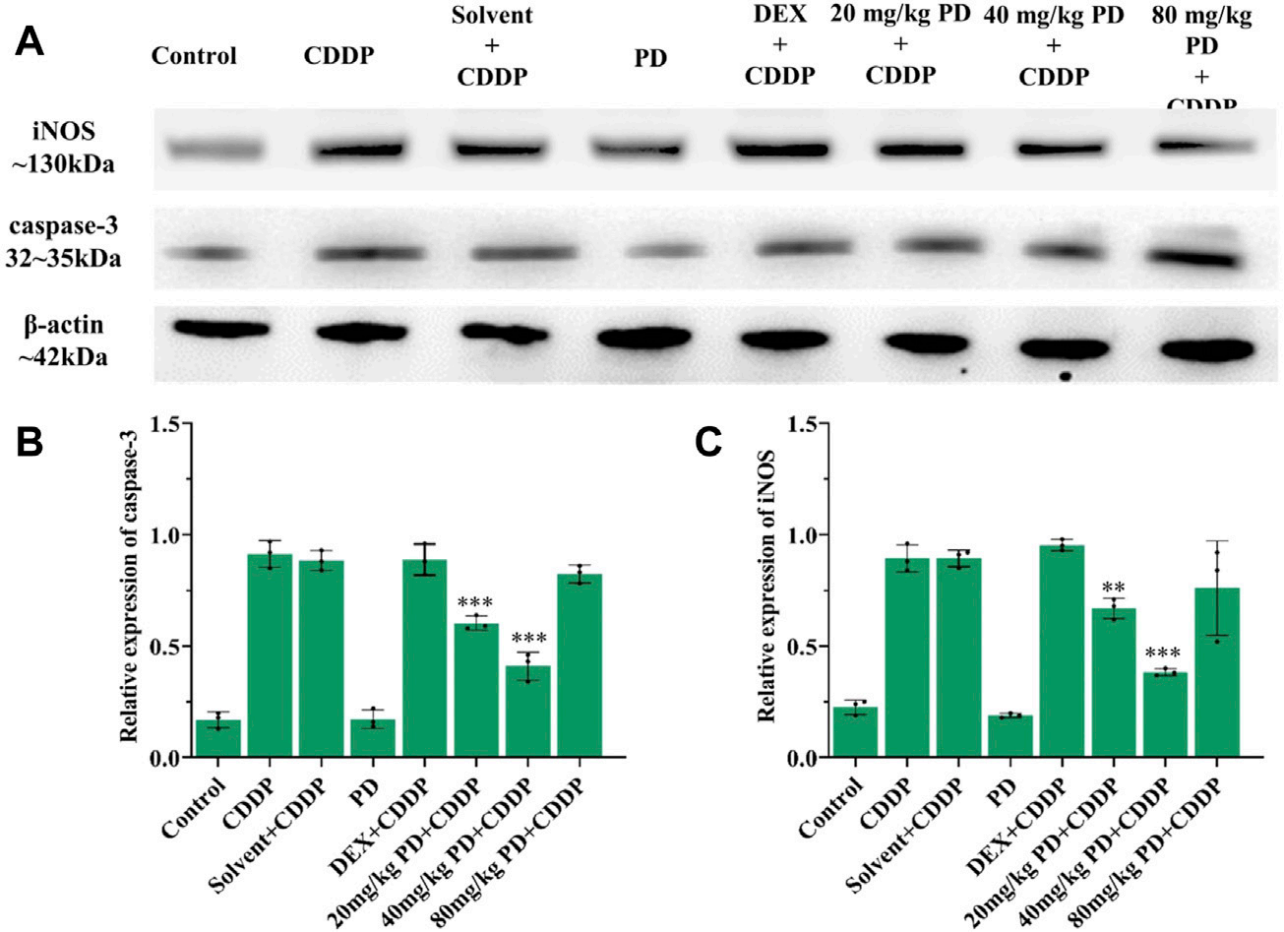
PD reduces cisplatin-induced cochlear oxidative damage. **(A)** The expression of iNOS and caspase-3 in each group after 3 days of drug administration. **(B)** and **(C)** PD administration before cisplatin decreased the expression of iNOS and caspase-3 in a concentration-dependent manner, presenting as PD (40 mg/kg) > PD (20 mg/kg) > PD (80 mg/kg). The effect of PD (40 mg/kg) was better than that of DEX (10 mg/kg, multi-dose) (*p <* 0.001). PD (40 mg/kg) injection alone did not cause oxidative stress damage and the increase of iNOS and caspase-3 expression in cochlea. *n* = 3. **p < 0.01, ***p < 0.001, all versus the CDDP group. Error bars represent mean ± SEM.

To verify the above results of immunostaining, we also performed immunoblotting analyses. The protein expression changes of Nrf2, HO-1, caspase-3 and iNOS from the cochleas of each group were consistent with the immunostaining results. Nrf2, HO-1, caspase-3 and iNOS were slightly expressed in the control group and PD (40 mg/kg) group. In the CDDP group and solvent + CDDP group, the expression of Nrf2 and HO-1 increased (Figure 9), accompanied by high expression of iNOS and caspase-3 (Figure 10). Compared with the CDDP group, the injection of PD before cisplatin administration significantly increased the expression of Nrf2 and HO-1, and the effect of PD (40 mg/kg) was the most significant (*p <* 0.001) (Figures 9A,B). In addition, the expression of iNOS and caspase-3 was significantly decreased after PD (40 mg/kg) administration (*p <* 0.001) (Figures 10A,B). The positive control group (DEX + CDDP) did not increase Nrf2, HO-1 expression or decrease iNOS and caspase-3 production compared with the CDDP group.

## Discussion

Sensorineural hearing loss is the most common type of hearing loss, including noise hearing loss, age-related hearing loss and drug-induced hearing loss (Cunningham and Tucci, 2017). These three factors can lead to hearing loss, and the accumulation of ROS in hair cells can be observed under pathological examination. Hair cell damage and loss caused by oxidative stress induced by ROS are considered to be an important reason for cisplatin-induced hearing loss (Gentilin et al., 2019). Reducing the generation of ROS after cisplatin treatment and improving the antioxidant capacity of cells are the key to the protection and treatment of cisplatin-induced hearing loss. In the preclinical and clinical studies on the treatment of cisplatin ototoxicity, several preventive agents are used to reduce the auditory function damage after cisplatin administration, including cisplatin inactivated agents, antioxidants, protective agents of antioxidant enzymes and anti-inflammatory drugs, unfortunately none of them can completely prevent or cure the hearing loss caused by cisplatin (Hazlitt et al., 2018). Seeking new, safer and more reliable drugs to prevent and mitigate cisplatin-induced hearing loss remains crucial and a challenge. Our study has found that after injection of cisplatin 12 mg/kg for 3 days, ABR threshold increased at 4, 8, 16, 24, and 32 kHz. While, after administration of PD, the hearing loss and threshold shift caused by cisplatin could be reduced. Most importantly, PD reduced OHCs loss by promoting Nrf2 and HO-1 expression and reducing iNOS and caspase-3 production.

In our animal model, cisplatin induced acute hearing loss 3 days after administration, presenting with increase of ABR thresholds and a large loss of OHCs, which was consistent with the reported results (Sun et al., 2015; Wang et al., 2018). Cisplatin ototoxicity is mainly manifested as hearing loss in the high frequency region, resulted from the loss of OHCs on the basilar membrane, the morphological abnormality, and loss of SGNs (Tabuchi et al., 2011). The damage of OHCs always starts from the basal turn of the cochlea and gradually develops to the apex, and the damage of OHCs are often earlier than that of inner hair cells, which is consistent with our H&E staining and basilar membrane paving results. We also observed that the loss of OHCs in the first row of the basilar membrane was more remarkable than that in the second and third rows (Figure 5). Some studies have demonstrated that OHCs and hair cells at the base exhibited stronger drug uptake capacity than IHCs and hair cells at the apex (Forge and Richardson, 1993; Kamimura et al., 1999), suggesting that the difference we observed in our study may be due to the different distribution of cation channels on the surface of hair cells (Lee et al., 2013). Other studies have reported that the content of reactive oxygen scavenger glutathione in apical hair cells was higher than that in basal hair cells, therefore, apical hair cells had stronger ability to resist oxidative stress injury than basal hair cells (Sha et al., 2001). In addition, some scholars believed that the concentrations of drugs in each cochlear turn might not be exactly the same, with high concentrations in the basal turn and the most serious damage to hair cells (Schacht et al., 2012). However, up to now, no conclusive agreement has been achieved and further study needs to be carried out to clarify this point (Lee et al., 2013). Our research team tends to recognize that hair cells in different parts have different ability to uptake drugs, resulting in different damage degrees. The uptake ability of OHCs is stronger than that of inner hair cells, and the uptake ability of OHCs in basal turn is more than that of apex. On the same basilar membrane, the OHCs in the first row were closer to the source of blood supply than those in the second and third rows, hence they uptook more drugs, leading to more significant damage.

PD is a natural active ingredient extracted from traditional Chinese medicinal plant polygonum cuspidatum. It has been widely acknowledged due to its anti-inflammatory, anti-oxidative stress, anti-ischemic injury, anti-atherosclerosis, anti-apoptosis, anti-tumor, antibacterial and antiviral effects (Sun and Wang, 2020; Wu et al., 2020). As the hydrolysate of PD, resveratrol has been reported to reduce cisplatin-induced ototoxicity. Intraperitoneal injection of resveratrol (100 mg/kg) reduced the increase of ABR threshold induced by cisplatin in rats (Simsek et al., 2013). Intraperitoneal administration of resveratrol 10 mg/kg for 5 days could reduce the decrease of distortion products induced by cisplatin in guinea pigs (Erdem et al., 2012). Unfortunately, the mechanism of resveratrol in improving cisplatin-induced hearing loss was not demonstrated. More importantly, PD has better antioxidant effect than resveratrol *in vivo* (Wang et al., 2015). However, the mechanism of PD in the treatment of hearing impairment was not reported. Our study found that intraperitoneal injection of PD 1 h before cisplatin administration could significantly reduce the increase of ABR threshold at 4, 8, 16, 24 kHz, while had little protective effect on high-frequency hearing (32 kHz) (Figure 1C). Moreover, the hearing protective effect of PD (40 mg/kg) was better than that of PD (20 mg/kg), while PD (80 mg/kg) showed no additional effect of hearing protection. In addition, PD (40 mg/kg) presented better effect of hearing protection than DEX (10 mg/kg, multiple-dose). In morphology, cisplatin (12 mg/kg) administration also resulted in the loss of a large number of OHCs and damage of static cilia on the basilar membrane of the cochlea, while PD (40 mg/kg) could significantly reduce the toxic effect of cisplatin on HCs (Figure 5), suggesting the potential ability of PD in reducing the ototoxicity of cisplatin.

To treat and alleviate cisplatin-induced hearing loss, it is essential to unravel the underlying mechanism of the effective antioxidant. Nrf2 is a transcription factor encoded by NFE2L2 gene and plays an important role in regulating redox homeostasis (Sykiotis and Bohmann, 2010). In humans and common domestic rats, NFE2L2 gene is located on chromosome 2. Nrf2 is expressed in cells of human and animal inner ear, liver, kidney, spleen, heart and other tissues (Cuadrado et al., 2019). Under normal physiological conditions, Nrf2 binds to Keap1 and anchors in the cytoplasm, followed by ubiquitination and rapid proteasome degradation. Under oxidative stress, ubiquitination of Nrf2 is blocked, allowing the newly synthesized Nrf2 to be transferred and aggregated into the nucleus (Itoh et al., 2004; Jung and Kwak, 2010; Baird and Dinkova-Kostova, 2011). After Nrf2 was translocated into the nucleus, it first formed heterodimers with small Maf protein, and then combined with antioxidant response element (ARE) to initiate the transcription of downstream antioxidant enzyme genes regulated by Nrf2. In our study, we have found that Nrf2 expression was low and mainly distributed in the cytoplasm in the absence of cisplatin and PD (Figures 8A,B). However, after cisplatin and PD administration, fluorescence signal of Nrf2 was observed in the nucleus (Figures 8C–F). This may suggest that PD can also promote the nuclear translocation of Nrf2 to increase the expression of antioxidant genes downstream of Nrf2. HO-1, a downstream product of Nrf2, exerts antioxidant, anti-apoptotic and anti-inflammatory effects through its products bilirubin/choline and carbon monoxide (Ma, 2013). HO-1 is considered to be an important antioxidant factor in cisplatin-induced oxidative stress injury after Nrf2-ARE signaling pathway activation in many studies (Ma et al., 2015; Fetoni et al., 2016; Youn et al., 2017). After administration of PD, the up-regulation of HO-1 expression in SGNs, OHCs and SV appears to be synchronized with the translocation in Nrf2 (Figures 7B, 8E,F). PD may reduce oxidative stress injury in the cochlea after cisplatin administration by activating Nrf2/HO-1 signaling pathway (Figures 7B, 8E,F), including reducing iNOS-induced NO production and apoptotic protein caspase-3 production, so as to protect hair cells from damage and improve cisplatin-induced hearing damage.

It should be noted that compared with single high-dose systemic injection or administration through circular window membrane in previous studies, three-cycle cisplatin administration is an ideal and clinically relevant animal model system for studying cisplatin-induced ototoxicity and screening otoprotective drugs *in vivo*. It increases the residence time of cisplatin in animal models, more similar to clinical regimens (Breglio et al., 2017). It is necessary to further study the protective effect of PD on hearing in the case of cisplatin-induced ototoxicity. More importantly, PD may play a hearing protection role not only by activating Nrf2-ARE signal axis, but also by interacting with other transcription factors in hearing loss. We are planning to investigate on those points in our future study. In addition, we need to consider whether the drug has a positive or negative effect on the efficacy of cisplatin in terms of hearing protection, tumor growth and systemic conditions. Our results in guinea pigs should be interpreted with more caution. The activation of Nrf2/HO-1 signaling pathway is an important way for PD to play a protective role in hearing. This study will be more convincing to observe the protective effect of PD after knocking out or inhibiting the expressions of Nrf2 and HO-1. This will be incorporated into our future studies. Nevertheless, our study revealed the potential of PD to play a protective role in hearing through the activation of Nrf2/HO-1 signaling pathway, but further assessment is necessary.

## Conclusion

Cisplatin often causes damage to OHCs and spiral ganglion cells, resulting in hearing loss. PD, as a natural extract of traditional Chinese medicine, can alleviate cisplatin-induced acute hearing loss. PD may activate the transcription and expression of HO-1 and other downstream antioxidant factors by promoting the nuclear translocation of Nrf2, thereby reducing oxidative stress injury in cochlea and reducing the apoptosis of OHCs and SGNs caused by NO and caspase-3. In summary, to our knowledge, it is first report to demonstrate the effect of PD on reducing cisplatin-induced hearing loss and its possible underlying mechanism, providing a new strategy for clinical prevention and treatment of cisplatin-induced hearing loss.

## Data Availability

The raw data supporting the conclusions of this article will be made available by the authors, without undue reservation.

## Abbreviations

ABR, auditory brainstem response; GSH-Px, glutathione peroxidase; HO-1, heme oxygenase-1; iNOS, inducible nitric oxide synthase; Nrf2, nuclear factor erythroid 2-related factor 2; OHCs, outer hair cells; PD, polydatin; ROS, reactive oxygen species; SGNs, spiral ganglion neurons; SOD, superoxide dismutase; SPL, sound pressure level; SV, stria vascularis.
