# Supplementary material for: Polydatin activates the Nrf2/HO-1 signaling pathway to protect cisplatin-induced hearing loss in guinea pigs
Source: Front Pharmacol. 2022 Aug 4;13:887833. doi: 10.3389/fphar.2022.887833 (PMC9386133; doi:10.3389/fphar.2022.887833)
Supplement: Supplementary file 1 [file DataSheet1.docx]

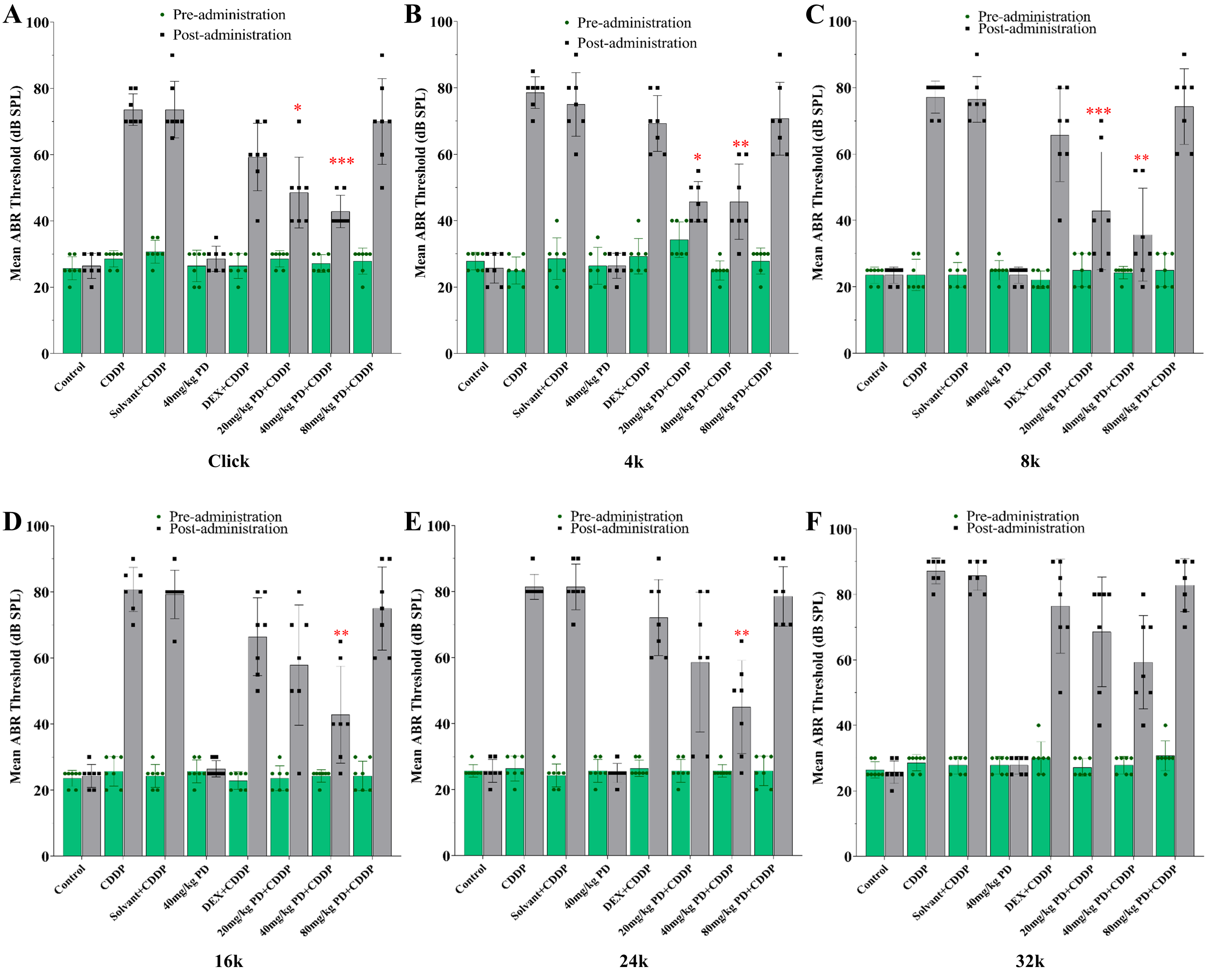


**Fig. S1. The mean ABR threshold of guinea pigs at each frequency in each group before and after drug administration.** n = 7. **P* < 0.05, ***P* < 0.01, and ****P* < 0.001, all versus the CDDP group. Error bars represent mean ± SEM.

**Measurement of oxidative stress and antioxidant enzyme activities in cochlea**

Four guinea pigs in each group were taken and immediately sacrificed after ABR test. The bilateral temporal bones of each guinea pig were quickly separated, and the auditory vesicles were opened to remove the cochlea. The cochlea was then quickly immersed in 4 ℃ Hanks solution. Remove the bony labyrinth under microscope and obtain the cochlear tissue. Appropriate amount of cochlea tissue samples were taken and homogenized in ice bath with PBS at 4 ℃. Then the homogenate was centrifuged at 4 ℃ and the supernatant was taken as the sample to detect the changes of ROS, RNS, NADPH oxidase, SOD1 and NQO1 in the cochlea.

Cisplatin induced cochlear oxidative stress damage including ROS and RNS generation. ROS content in cochlea was measured using a tissue ROS test kit (DHE) (HR8821, Baiaolaibo, China) according to the manufacturer’s protocol. Briefly, the homogenate supernatant of cochlea tissue was incubated with DHE at 37 ℃ for 30 min in dark. The fluorescence intensity of the homogenate supernatant was measured at the excitation wavelength of 488 nm and the emission wavelength of 610 nm using a microplate reader (Cytation 5, BioTek, America). Tissue RNS test kit (052) (HR8821, Baiaolaibo, China) was used to measure the content of RNS in the cochlea, and the procedure was similar to the above ROS test. The homogenate supernatant of cochlea tissue was incubated with BBoxiProbe® O52 at 37 ℃ for 30 min in dark. The fluorescence intensity of the homogenate supernatant was measured at the excitation wavelength of 488 nm and the emission wavelength of 530 nm using a microplate reader (Cytation 5, BioTek, America).

NADPH, as a major provider of subcellular reduction equivalents, can be oxidized to NADP^+^. Therefore, NADP^+^/NADPH is commonly used to react to the activation of NADPH oxidase and subsequent ROS production. The supernatant of the cochlea sample was taken and placed in a water bath at 60 ℃ for 30 min, then added with G6PDH solution, fully mixed and incubated in dark at 37 ℃ for 10 min. Add the chromogenic solution to the supernatant and mix, incubated in dark at 37 ℃ for 10 ~ 20 minutes. After the orange-yellow formazan was formed, the absorbance at 450 nm was measured by a microplate reader.


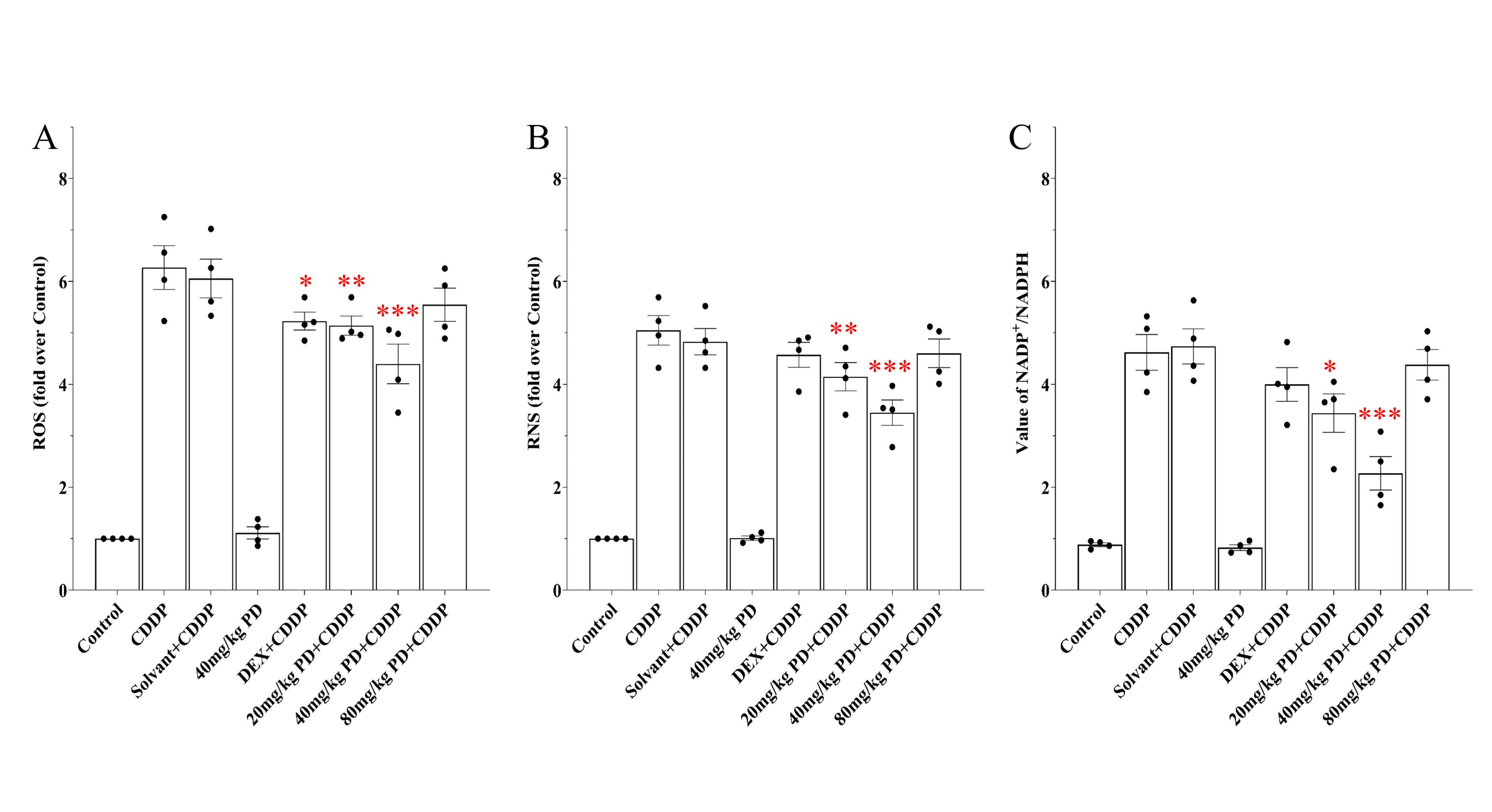


**Fig. S2. PD reduced ROS and RNS production and reduced the activity of NADPH oxidase in cisplatin-induced oxidative stress injury of cochlea.** (A) Compared with CDDP group, DEX, PD (20mg/kg) and PD (40mg/kg) all reduced the production of ROS in cochlea, with PD (40mg/kg) the best(*P =* 0.0001). (B) Compared with CDDP group, PD (20mg/kg) and PD (40mg/kg) all reduced the production of ROS in cochlea, with PD (40mg/kg) the best (*P <* 0.0001). (C) The activity of NADPH oxidase was indirectly evaluated by measuring the ratio of NADP^+^ to NADPH. Compared with CDDP group, PD (20mg/kg) and PD (40mg/kg) all reduced the activity of NADPH oxidase in cochlea, with PD (40mg/kg) the best (*P <* 0.0001). n = 4. **P < 0.05,* ***P <* 0.01, ****P <* 0.001, all versus the CDDP group. Error bars represent mean ± SEM.

The SOD1 activity of cochlea was determined using a SOD1 assay kit with WST-8 (S0103, Beyotime, China), according to the manufacturer’s protocol. After incubation with WST-8 at 37 ℃ for 30 min, the absorbance of the homogenate supernatant at 450 nm was measured using a microplate reader (Cytation 5, BioTek). The NQO1 activity of cochlea was determined using a NQO1 activity assay kit (ab184867, Abcam, England). The detection steps strictly follow the manufacturer’s protocol. The absorbance of the homogenate supernatant at 450 nm was measured using a microplate reader (Cytation 5, BioTek).


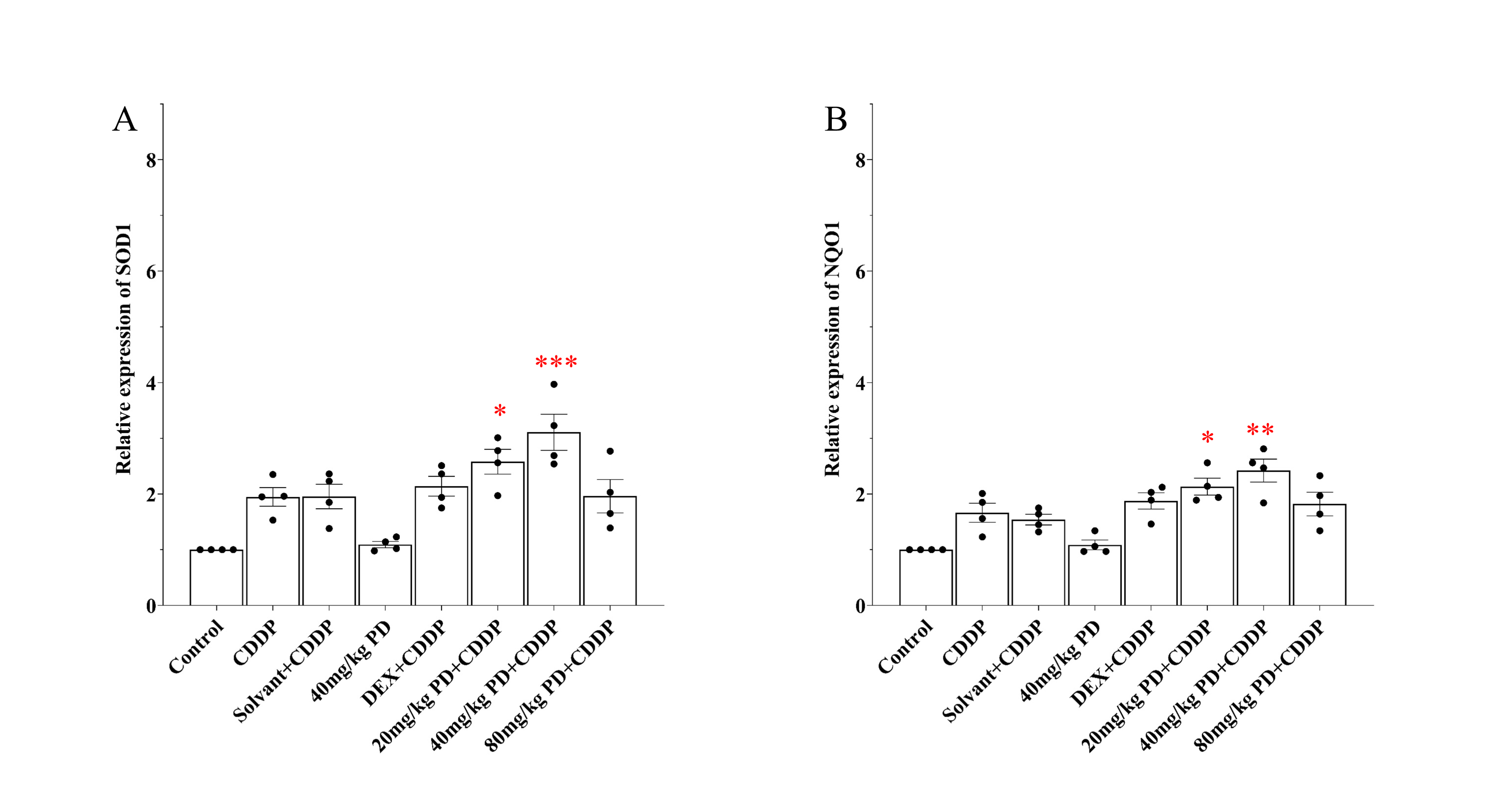


**Fig. S3. PD promoted the expression of downstream antioxidant factors of Nrf2 such as SOD1 and NQO1.** (A) Compared with CDDP group, DEX, PD (20mg/kg) and PD (40mg/kg) all improved the expression of SOD1 in cochlea, with PD (40mg/kg) the best (*P =* 0.0008). (B) Compared with CDDP group, PD (20mg/kg) and PD (40mg/kg) all improved the activity of NQO1 in cochlea, with PD (40mg/kg) the best (*P =* 0.0028). n = 4. **P < 0.05,* ***P <* 0.01, ****P <* 0.001, all versus the CDDP group. Error bars represent mean ± SEM.
